# Long-term follow-up with Granulocyte and Monocyte Apheresis re-treatment in patients with chronically active inflammatory bowel disease

**DOI:** 10.1186/1471-230X-10-73

**Published:** 2010-07-06

**Authors:** Annelie Lindberg, Michael Eberhardson, Mats Karlsson, Per Karlén

**Affiliations:** 1 Department of Clinical Science and Education, Karolinska Institutet, Södersjukhuset SE-118 83 Stockholm, Sweden

## Abstract

**Background:**

Patients with IBD and chronic inflammation refractory to conventional therapy often demonstrate higher risk of serious complications. Combinations of immunosuppression and biological treatment as well as surgical intervention are often used in this patient group. Hence, there is need for additional treatment options. In this observational study, focused on re-treatment and long-term results, Granulocyte/Monocyte Adsorption (GMA, Adacolumn^®^) treatment has been investigated to study efficacy, safety and quality of life in IBD-patients with chronic activity.

**Methods:**

Fifteen patients with ulcerative colitis and 25 patients with Crohn's disease, both groups with chronically active inflammation refractory to conventional medication were included in this observational study. The patients received 5-10 GMA sessions, and the clinical activity was assessed at baseline, after each completed course, and at week 10 and 20 by disease activity index, endoscopy and quality of life evaluation. Relapsed patients were re-treated by GMA in this follow-up study up to 58 months.

**Results:**

Clinical response was seen in 85% and complete remission in 65% of the patients. Ten patients in the UC-group (66%) and 16 patients in the CD-group (64%) maintained clinical and endoscopic remission for an average of 14 months. Fourteen patients who relapsed after showing initial remission were re-treated with GMA and 13 (93%) went into a second remission. Following further relapses, all of seven patients were successfully re-treated for the third time, all of three patients for the fourth time and one for a fifth time.

**Conclusions:**

IBD-patients with chronic inflammation despite conventional therapy seem to benefit from GMA. Re-treatment of relapsing remission patients seems to be effective.

## Background

Inflammatory bowel diseases (IBD), divided into the two main entities ulcerative colitis (UC) and Crohn's disease (CD), is a debilitating chronic inflammation of the intestine. A broad range of pharmacological therapies is available for treating IBD, including 5-aminosalicylate preparations, corticosteroids, and immunosuppressants (azathioprine/6-mercaptopurine). However, these treatments are hampered by lack of response in some patients and side effects may add to the disease complications [1,2]. Furthermore, in recent years, monoclonal antibodies to TNF-α (Tumor Necrosis Factor) have been approved for both CD [3,4], including maintenance [5], and UC [6-8], in patients who are unresponsive to the aforementioned baseline conventional medications. These biologics are associated with side effects such as opportunistic infections, tuberculosis, demyelinating syndromes, lymphoma and rarely also mortality [9-11]. In addition all immunosuppressive therapies, especially when used in combination, are associated with increased risk of opportunistic infections [12]. Another option for drug-refractory patients is surgery, an intervention associated with increased morbidity and disability [13,14]. The treatment-related complications tend to have substantial impact on patients health-related quality of life (HRQoL) [15-17]. In addition, according to a newly published report from the IBSEN study, the subgroups of patients who display chronic inflammation despite treatment reach 6% of UC- [18], and 19% of the CD-patients [19]. Another important health-related quality of life aspect concerns disease activity. Lix and co-authors recently showed that one-third of the participants with perpetual inflammation demonstrate significantly lower improvement in disease-specific QoL compared to patients with fluctuating activity [20]. Hence, there is a need for effective and well-tolerated therapies for IBD-patients with chronic inflammation who are unresponsive or intolerant to first-line medications and biologics.

IBD-patients are characterised by elevated levels of granulocytes and monocytes in peripheral blood, circulating immune complexes, and leukocyte-derived inflammatory factors like cytokines and chemokines [21-24]. A major mechanism behind IBD seems to be over-activated intestinal immune response against luminal antigen(s). Accordingly, in active IBD, the mucosa is infiltrated with large numbers of granulocytes, lymphocytes, plasma cells, and macrophages. These immunocompetent cells produce cytokines such as TNF-a, IL-1-b, IL-6, IL- 8, IL-12, IL-23, and IFN-g, which further stimulate the local inflammation [25-29]. Indeed, histological examinations of mucosal biopsies from patients with active IBD reveal a spectrum of pathologic manifestations among which the abundance of neutrophils relates specifically to clinical activity and severity of the disease [30]. Since cytokines from activated granulocytes have a validated role in the immunopathogenesis of IBD and the fact that circulating monocytes are recruited for antigen-presenting functions in the inflammation [26-28,31], these cells appear as logical targets for down-regulating the inflammation. Granulocyte/Monocyte Adsorption (GMA) constitutes an extracorporeal perfusion of the blood through a column filled with cellulose acetate beads binding the aforementioned immune cells to the matrix. The anti-inflammatory effect of GMA is supported by a recently published meta-analysis [32]. Therefore, it appears interesting to apply selective granulocyte and monocyte adsorption (Adacolumn^®^) to patients with chronically active IBD refractory to conventional medication.

This open-label, observational study was set out to investigate efficacy, safety and quality of life in patients with chronic activity in IBD with focus on re-treatment strategy with GMA and long-term follow-up.

## Methods

### Ethical considerations

The Adacolumn is CE marked with regulatory approval in all countries of the EU territories. All patient data was dealt with in an anonymous way. This study was approved by the regional ethical committee of Stockholm (Dnr: 2009/367-31/2). Signed informed consent was obtained from all patients.

### Device description

Granulocyte/monocyte apheresis (GMA) with Adacolumn^® ^(Otsuka, London, UK) is an extracorporeal device. The apheresis column (about 20 cm long × 9 cm in diameter poly-carbonate cylinder) contains specially designed cellulose acetate beads (35 000 beads), each with an average diameter of 2 mm, in physiological saline buffer [33]. The column has a capacity of 335 ml. Adacolumn^® ^is connected to the patient by two venous blood lines and blood is circulated through the column with the help of a pump (Adamonitor). Before start, the system is primed by physiological saline containing an anticoagulant (Heparin, 5000 IE in 1000 ml of physiological saline) and during extracorporeal circulation Heparin is continually added, (100IE/ml, 15 ml/hour is used). The apheresis procedure lasts for 60 minutes and the system forms an extracorporeal circuit interposed between two veins, preferentially in the antecubital fossae using 1,3 mm Venflon^® ^needles. As blood passes through the column, granulocytes and monocytes are selectively adsorbed to the beads [24,33]. In contrast, lymphocytes and erythrocytes do not adhere to the matrix [24,34], and return to the patient via the column outflow line. This selective adsorption of activated granulocytes and monocytes is expected to reduce the inflammatory activity associated with TNF-α, IL-1-b, IL-6, IL- 8 and IL23 [25-29]. The adsorbed cells are rapidly replaced by mobilisation of inactive leukocytes, i.e. CD10 ‾ neutrophils and HLA-DR (low) down-regulated monocytes from the bone marrow [34].

### Patients

This observational study was carried out in an open Gastroenterology ward at South Hospital in Stockholm, from April 2002 to December 2006. IBD-patients with chronic inflammation in spite of conventional medication were consecutively recruited and prospectively registered. All 40 eligible patients accepted GMA treatment. The ulcerative colitis patients were assessed by the UC disease activity index UC-DAI [35] and the Crohn's disease patients were assessed by Harvey Bradshaw Index (HBI) [36]. Forty patients were treated with GMA therapy: 15 chronically active UC-patients with mean disease activity index (UC-DAI) of 8,1 (range 4-9) and 25 chronically active CD-patients with mean HBI of 12,5 (range 5-27). UC-DAI and HBI were initiated from the second patient. Chronic activity was defined as at least six months duration without remission in spite of conventional treatment in optimal time and dosage (corticosteroids, 5-aminosalicylates, azathioprine or 6-mercaptopurine). In addition, all CD patients were offered infliximab treatment but 16 declined due to concern over side-effects. The patients received weekly GMA sessions for a period of 5-10 weeks (mean 8 weeks). Initially five sessions were administrated to all patients followed by evaluation. In case of non-response 1-3 additional sessions were given.

All treatments were provided by two GMA-trained and experienced IBD nurses. Before GMA, the current vessels were pre-treated with EMLA^® ^-plaster (local anaesthetic with lidokain and prilokain) to decrease rupture and inflammation of the veins. The patients were also instructed to appear warmly dressed to diminish problems with venous access and to be well hydrated to avoid headache during or after treatment. The patients' demographic characteristics, previous medication, disease location, smoking status and activity are presented in Table 1. This cohort of 40 patients represents a sub-group of patient's refractory to conventional therapy. Noteworthy, 36% of the included CD-patients were non-responders to prior infliximab treatment. Twenty-five patients were treated with concomitant Azathioprine and 18 patients were medicated with 5-ASA at stable doses throughout treatment and follow up. Seven patients achieving remission in this study were given tapering systemic Prednisolone (median dose 17,5 mg, range 5-40 mg) at start of the apheresis treatment. All patients were free from systemic corticosteroids at end of treatment.

**Table 1:** Patient characteristics

	UC	CD
N	15	25

Gender (n)		
Female	4	11
Male	11	14

Age median (range)	25 (16-57)	33 (18-51)

Disease duration (y) (median, range)	1-40 (10,7)	1-25 (9,6)

Smokers	0	0

Disease severity (UCDAI, HB) (n)		
Mild	1	
Moderate	13	
Severe		
>5 in HB (range)		24 (5-27)

Previous medication (n)		
Aminosalicylates	15	25
Glucocorticosteroids	15	25
Immunomodulators (6-MP/AZA)	15	25
Infliximab		9
Antibiotics	12	23

Patients with extraintestinal manifestations (n)	4	12

Patients with previous abdominal surgery for IBD (n)	4	6

Patients with perianal fistulas (n)		5

Disease location		
Extensive	6	
Left side	7	
Proctitis	2	
Extensive colonic		21
Small bowel + colonic		1
Esofagus + small bowel		1
Stomach + small bowel + colonic		1

**UCDAI **score of 3 to 6 is defined as mild, 7 to 10 as moderate and 11 to 12 as severe UC.

**HB score **≥ 5 defined as active CD

### Evaluation

Efficacy assessments were performed after each completed GMA course and then at week 10 and 20 post-treatment. Response was classified according to three categories: 0 = no change or worsening of symptoms; 1 = partial response: improvement or tapering of steroids without worsening; 2 = complete remission, absence, or near absence of all clinical symptoms without an increase in steroid dose [37]. In addition, endoscopic assessment (at least flexible sigmoidoscopy) was performed in all colonic disease (UC and CD) patients according to UC-DAI. Normal mucosa (mucosal appearance = 0) was classified as complete remission and erythema and decreased vascular pattern was classified as response (mucosal appearance = 1). Patients with mucosal appearance >1 were classified as non-responders. Patients who achieved remission within 20 weeks were monitored clinically and endoscopically every third month. HRQoL measurement by Short Health Scale (SHS) was initiated from the sixth patient in the study. SHS is a validated four-item self-administrated disease-specific questionnaire that includes major health dimensions such as symptom burden, function, disease-related worry and general well-being. The responses were scored on a 100 mm visual analogue scale [38,39].

### Re-treatment

During follow-up time, all patients were instructed to inform the study team concerning significant changes in their disease status. Relapsing patients were re-treated with GMA.

### Statistical analysis

The levels of SHS sub-scales and the scores according to HBI and UC-DAI are shown in box plots, one box per time point measured. Boxes show interquartile range at each time with median presented in bold. Differences in median score between time points were tested by the Mann-Whitney test, p-values presented in the figure. The laboratory measurements were tested for differences between groups by Kruskal-Wallis test (for Haemoglobin, White cell count and Albumin) and by χ2-test (for C-reactive protein). Analyses and graphs were performed in R 2.7.2 and a p-value of 0.05 or less was considered significant.

The differences in survival curves were tested with log rank test and were performed in PASW statistics 18.

## Results

### Clinical efficacy

**Figure 1:**
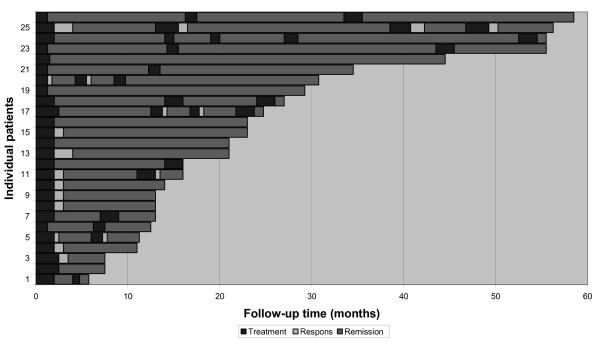
**Individual IBD patients with chronic active disease refractory to conventional medications**. Patients in clinical and endoscopic remission secondary to selective apheresis treatment. Follow-up time in months.

**Figure 2:**
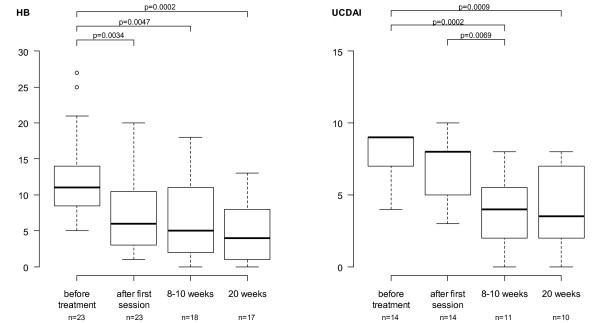
**Scores on Harvey Bradshaw Index (HB) and Ulcerative Colitis Disease Activity Index (UCDAI) in boxplots**. Boxes show interquartile range at each time. Median in bold. Differences in median score between time points were tested by the Mann-Whitney test, p-values presented. P-value ≤ 0,05 was considered significant HB score >5 is defined as active disease. UCDAI score of 3 to 6 is defined as mild, 7 to 10 as moderate and 11 to 12 as severe disease

A total of 453 GMA procedures were performed and adequate venous access was achieved in all patients. Thus, no patients were excluded due to inadequate venous access. Out of 40 patients with chronically inflamed mucosa (15 with UC and 25 with CD) and refractory to conventional medications, 34 (85%) responded to GMA. Furthermore, 26 patients, ten with UC and 16 with CD (65%) achieved clinical as well as endoscopic remission for an average of 14 months, ranging from two to 58 months (Figure 1). The laboratory data are summarised in Table 2. HBI- and UC-DAI-outcome, significant when assessed between baseline and 20 weeks follow-up, are displayed as box plots in Figure 2. Data from three patients are missing (UC-DAI and HBI were initiated from the second patient and one CD-patient had ileostoma).

**Table 2:** Laboratory data

	Before treatment	10 weeks after treatment	20 weeks after treatment
**Remission patients (n = 26)**			
Haemoglobin Mean g/l (range g/l)	135 (97-164)	134 (95-154)	137 (117-162)
Mean white cell count x10/1 (range)	7.8 (1.8-15.6)	7.1 (2.4-15.8)	6.8 (0.0-12.6)
Albumin Mean g/l (range g/l)	39.4 (33.0-50.0)	39.8 (34.0-45.0)	40.3 (31.0-48.0)
CRP N 10 mg/l or over (total)	3 (23)	3 (23)	2 (22)
**Responded patients (n = 8)**			
Haemoglobin Mean g/l (range g/l)	135 (100-162)	127 (107-156)	130 (113-150)
Mean white cell count x10/1 (range)	12.5 (9.7-17.2)	8.9 (6.0-11.3)	8.6 (3.8-10.8)
Albumin Mean g/l (range g/l)	34.8 (29.0-41.0)	37.6 (33.0-42.0)	36.7 (32.0-39.0)
CRP N 10 mg/l or over (total)	3 (8)	2 (7)	3 (7)
**Non response patients (n = 6)**			
Haemoglobin Mean g/l (range g/l)	120 (82-164)	122 (93-146)	131 (112-153)
Mean white cell count x10/1 (range)	8.9 (4.8-24.3)	8.3 (5.4-19.7)	10.5 (4.6-21.6)
Albumin Mean g/l (range g/l)	39.0 (35.0-42.0)	37.3 (32.0-41.0)	37.5 (34.0-41.0)
CRP N 10 mg/l or over (total)	3 (6)	2 (6)	3 (5)

Before treatment only white cell count differed significantly (p = 0.004) between the three groups (remission/response/non-responders). At 20 weeks after treatment, however, as white cell count still differed significantly between the groups (p = 0.038), so did Albumin (p = 0.038) and C-Reactive Protein (p = 0.021).

Among patients that achieved remission eleven maintained azathioprin and ten amonosalicylates throughout treatment and follow up.

### GMA-treatment of relapsed patients

During the follow-up time 14 out of 26 (ten CD and four UC) patients, who initially achieved clinical and endoscopic remission after GMA-treatment, relapsed. These patients were re-treated with GMA, which resulted in 13 (93%) patients achieving a second remission. Following further relapses, all of seven patients were successfully re-treated for the third time, all of three patients for the fourth time and one patient for a fifth time. Thus, the remission rates after the first, second, third fourth and fifth course of GMA treatment were 65%, 93%, 100%, 100% and 100%, respectively (albeit in small groups of patients). All 26 patients who initially achieved remission became corticosteroid-free during the follow-up period. Other concomitant medications at inclusion were stable throughout follow-up. However, two patients deteriorated in their second and fourth re-treatment. One of these patients went into remission after additional intravenous corticosteroids and the other patient reached remission after infliximab treatment and continued on that medication. Three patients who achieved remission were retreated with corticosteroids as rescue therapy for relapse due to temporary limited access of Adacolumns. Additional therapy during the GMA treatment and follow-up was limited to these patients.

**Figure 3:**
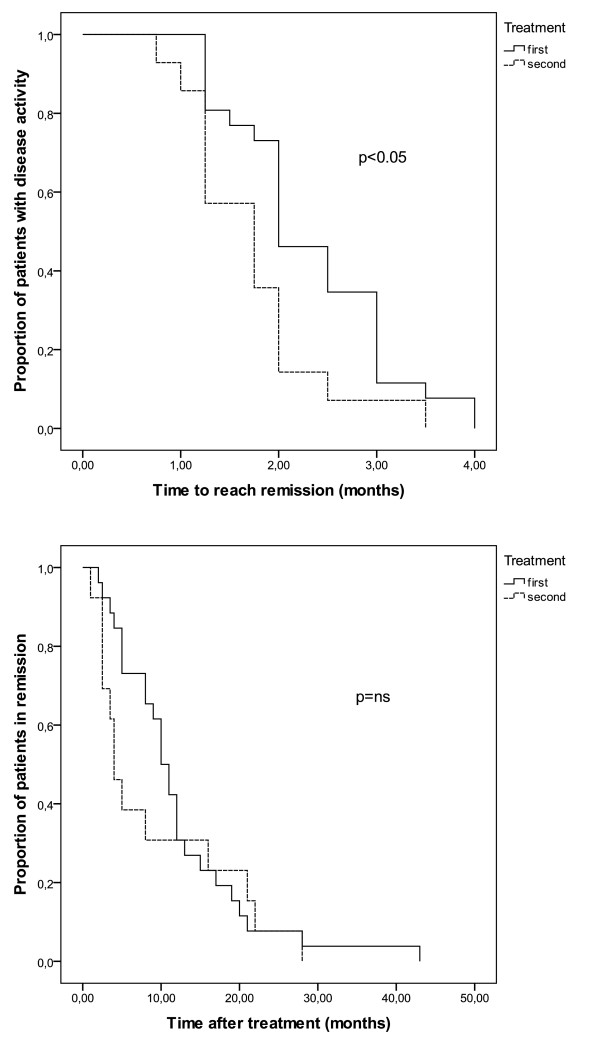
**Proportion of patients achieving remission and proportion of patients staying in remission over time**.

Proportion of patients achieving remission and proportion of patients staying in remission over time are shown in Figure 3. Remissions are achieved significantly faster after the second treatment (p = 0.015).

### Quality of life assessment

**Figure 4:**
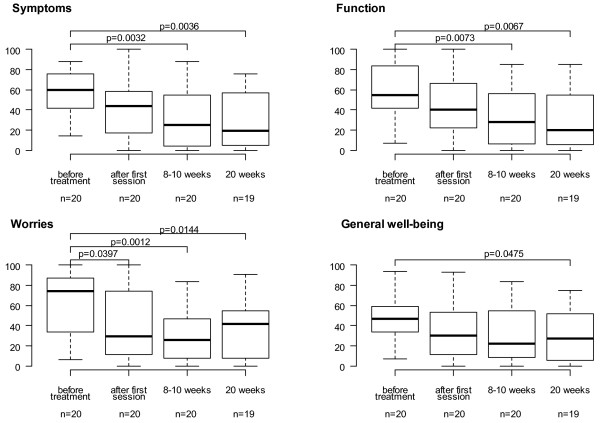
**The levels of subscales of the Short Health Scale (SHS) in boxplots , one box per time point measured**. Boxes show interquartile range at each time, median in bold. Differences in median score between time points were tested by the Mann-Whitney test. P-value ≤ 0,05 was considered significant.

HRQoL scoring evaluated according to SHS was significantly increased in all sub-groups assessed from baseline to week 20 as presented in Figure 4.

### Safety and tolerability

The GMA treatment was well tolerated. No serious side effects related to GMA were observed. Two patients developed transient headache, one person severe, and four patients exhibited transient fatigue. No patients discontinued the GMA therapy; thus compliance reached 100% in the investigated.

## Discussion

Studies of immunopathogenesis behind IBD have recently identified the innate immune system as a major player in mucosal inflammation [40]. The activated granulocytes and macrophages are important sources of pro-inflammatory cytokines [21,25,31]. Moreover, some of the pro-inflammatory monocytes are known to develop into locally infiltrating dendritic cells, which are professional antigen-presenting cells that maintain the mucosal inflammation by continuous stimulation of naïve T cells [41,42]. In this context, the removal of these cells by leukocytapheresis provides us with a treatment strategy that targets major catalysts behind the intestinal inflammation. Furthermore, GMA (Adacolumn^®^) has been shown to improve clinical symptoms in patients with UC [22,24,43] and CD [44,45]. GMA treatment has also been suggested to significantly reduce the dosage of prednisolone required to relieve symptoms in active UC-patients [46]. A pilot study by Bresci et al even implies that GMA is more effective with less frequent and serious side effects than prednisolone in acute UC [47]. Maiden and co-workers recently confirmed the efficacy of Adacolumn apheresis treatment in reducing relapse rates in UC as well as CD patients [48]. However, to our knowledge the present study is the first to elucidate the effect of GMA-treatment in long-term follow-up including re-treatment at relapse.

The consecutive recruitment of patients with chronic inflammation resulted in a UC/CD ratio of 3/5 which may reflect the prevalence [18,19]. The relatively small proportion of CD patients with small bowel involvement in this study, 3/25 (12%) compared to expected 48% in Stockholm County [49], may indicate a higher risk for CD patients with colonic inflammation to become chronic. However this reflection remains speculative within the scope of this report. An explanation to this recruitment discrepancy might also be a preference to refer patients with chronic inflammation localized to the small bowel or ileo-caecal region to limited surgery while trying GMA treatment on the colitis patients before colectomy.

This study includes a cohort of refractory patients who were re-treated with GMA upon relapse. We found that close to 100% of the patients who initially responded to a course of GMA also responded to subsequent GMA re-treatments. This may serve as a guide to select responders in the light of the high cost of this treatment.

In the present study including, 40 patients with chronically active IBD (15 with UC and 25 with CD) refractory to conventional medications, 34 patients (85%) responded to GMA. Further, 26 patients (65%) remained in clinical as well as endoscopic remission for an average of 14 months, ranging from two to 58 months. These remission values (65%) are in line with other studies, however with more homogenous patient selection [45,50]. All 26 patients remained steroid-free during the follow-up period. Hence, GMA implicated substantial steroid-sparing effects. During the follow-up time, patients with relapsed disease were re-treated with GMA, resulting in high remission rates after the second to fourth time of GMA treatment. This finding indicated that maintenance therapy would be an interesting option but in lack of evidence at that time such a regime was not regarded as appropriate. Evidence for maintenance therapy has however recently been shown [48].

These encouraging results must be critically judged in the light of the open uncontrolled study design. Extracorporeal treatment may hold substantial placebo effects, even more pronounced than seen in similar patients groups [51]. However, the patients included in this study were chronically inflamed and refractory to prior conventional treatments and almost all first-time responders who relapsed during the study period reached remission following re-treatment. The expected remission rate in one year in this sub-group of patients is unknown. The 65% clinical and endoscopic remission rate noted in the present group is in contrast to the placebo-controlled and randomised study [52] which was unsuccessful in reaching significant treatment effect. However, the placebo-controlled study included a more heterogeneous group of IBD-patients regarding disease patterns. In addition, the follow-up time only reached two to three weeks after completed GMA course. The discrepancy between the outcome of the current study and the aforementioned placebo-controlled study might possibly be explained by the presumption that chronically inflamed patients represent a more homogenous immunological phenotype regarding the importance of monocytes for driving the inflammation.

A total of 453 GMA procedures were performed and adequate venous access was achieved in all patients. Therefore, no exclusion of patients due to inadequate venous access was reported. Unlike other studies [53], we could overcome the problems with venous access partly by ensuring good peripheral blood flow and partly by using non-traumatising needles. The GMA treatment was well tolerated without serious side effect related to the apheresis procedure. Two patients developed transient headache, one person severe, and four patients showed transient fatigue. No patient discontinued the GMA treatment, which resulted in a compliance of 100%. HQoL were significantly increased in all sub-groups assessed between baseline and week 20. This is promising since the consistent active patients seem to reach lower quality of life scoring compared to other sub-groups [20]. However, the dimensions "worry" and to a lesser extent even "general well being" in SHS increased assessed between the later time points measured. The impending fact that the close caring and weekly monitoring of these patients was reaching a final stage could partly explain these findings, in spite of the good contact availableness to the IBD nurses in continuation.

A total of 323 GMA sessions were given to the remission patients upon relapse and a total of 566 months in remission was achieved, equivalent to 1.75 months per apheresis column for patients in remission, which reflects a cost of 1150 $/remission month.

All 40 patients in the study suffered from chronic inflammation, defined as at least six months without remission in spite of conventional medication including immunosuppressors. Taking the pre-treatment clinical activity into account, each patient may be regarded as her or his own control. The continuous time period with active IBD prior to entry does not suggest that the GMA-induced remission correlates with the natural course of the disease in these chronically debilitated patients. Nonetheless, the observations in this relatively small cohort of IBD patients need to be further investigated in prospective, randomised controlled trials including larger cohorts of IBD-patients.

A sub-group of patients with IBD is characterised by a chronically activated immune response with a persistently inflamed mucosa. Defects in the innate immune response may be a major contributor to the onset of the inflammation, reflected by continuous activation of granulocytes and monocytes [40]. These cells are crucial sources for the pro-inflammatory cytokines seen in the intestinal lesions [31], and many of the circulating monocytes are targeted for antigen-presenting functions at the inflammatory site[31]. Hence, the removal of these leukocytes by extracorporeal selective adsorption may be an efficient approach to down-regulate the immune response in patients with limited effects from conventional medication.

## Conclusions

85% of the drug-refractory patients responded to GMA. The remission rate among relapsing patients was close to 100%. Technical feasibility and patient's tolerability were excellent. These finding may indicate that scheduled therapy with GMA should be evaluated in IBD patients with chronic disease activity who respond to an initial GMA course.

## Competing interests

All authors have lectured on behalf of the Otsuka Pharmaceutical Scandinavia and received honoraria. Otsuka Pharmaceutical Scandinavia has given financial support to the statistical analysis performed by an independent statistical company (Statisticon AB, Wallingatan 38, SE-111 24 Stockholm, Sweden). Otsuka company had no role in study design, data collection and analysis, decision to publish, or preparation of the manuscript.

## Authors' contributions

AL and PK conceptualized and designed the study, analyzed and interpreted the data and wrote the manuscript. AL has performed the GMA treatments. ME assisted in the analysis and interpretation and critically revised the manuscript. MK assisted in the analysis and interpretation of data. All authors read and approved the final version of the manuscript.

## Pre-publication history

The pre-publication history for this paper can be accessed here:

http://www.biomedcentral.com/1471-230X/10/73/prepub
